# Identification of Candidate Serum Biomarkers for *Schistosoma mansoni* Infected Mice Using Multiple Proteomic Platforms

**DOI:** 10.1371/journal.pone.0154465

**Published:** 2016-05-03

**Authors:** Manal I. Kardoush, Brian J. Ward, Momar Ndao

**Affiliations:** 1 Institute of Parasitology, McGill University, Montreal, Quebec, Canada; 2 National Reference Centre for Parasitology, Research Institute of the McGill University Health Centre, Montreal, Quebec, Canada; 3 Department of Parasitology, Faculty of medicine, Benha University, Benha, Qalubia, Egypt; 4 JD MacLean Tropical Diseases Centre, the McGill University Health Centre, Montreal, Quebec, Canada; Alexandria University, EGYPT

## Abstract

**Background:**

Schistosomiasis is an important helminth infection of humans. There are few reliable diagnostic biomarkers for early infection, for recurrent infection or to document successful treatment. In this study, we compared serum protein profiles in uninfected and infected mice to identify disease stage-specific biomarkers.

**Methods:**

Serum collected from CD1 mice infected with 50–200 *Schistosoma mansoni* cercariae were analyzed before infection and at 3, 6 and 12 weeks post-infection using three mass spectrometric (MS) platforms.

**Results:**

Using SELDI-TOF MS, 66 discriminating m/z peaks were detected between *S*. *mansoni* infected mice and healthy controls. Used in various combinations, these peaks could 1) reliably diagnose early-stage disease, 2) distinguish between acute and chronic infection and 3) diagnose *S*. *mansoni* infection regardless the parasite burden. The most important contributors to these diagnostic algorithms were peaks at 3.7, 13 and 46 kDa. Employing sample fractionation and differential gel electrophoresis, we analyzed gel slices either by MALDI-TOF MS or Velos Orbitrap MS. The former yielded eight differentially-expressed host proteins in the serum at different disease stages including transferrin and alpha 1- antitrypsin. The latter suggested the presence of a surprising number of parasite-origin proteins in the serum during both the acute (n = 200) and chronic (n = 105) stages. The Orbitrap platform also identified many differentially-expressed host-origin serum proteins during the acute and chronic stages (296 and 220 respectively). The presence of one of the schistosome proteins, glutathione S transferase (GST: 25 KDa), was confirmed by Western Blot. This study provides proof-of-principle for an approach that can yield a large number of novel candidate biomarkers for Schistosoma infection.

## Introduction

Schistosomiasis is a public health problem of global importance [1]. For both surveillance and the optimal treatment of patients, rapid and sensitive diagnostic tests are needed that can detect infection soon after exposure and when parasite burden is low. Although the current gold-standard test for *Schistosoma mansoni* is microscopic detection of the eggs in stool, eggs first appear only 6–8 weeks after infection. This method also has poor sensitivity when few parasites are present and during the chronic stage of infection when the passage of eggs is typically low [2]. Other tools used to diagnose and monitor schistosomiasis include the detection of circulating antigens or antibodies and ultrasound to assess liver fibrosis and hepatosplenomegaly [3], [4], [5]. Polymerase chain reaction (PCR) has been used to detect *S*. *mansoni* DNA in human fecal samples [6], [7]. All of these tests have important limitations related to their complexity, expense, sensitivity and/or cross-reactivity with other helminth infections. Most cannot discriminate between active and past infections [8], [9]. Therefore, there is a need for new schistosomiasis diagnostic options.

Mass spectrometry (MS) has the potential to modernize *S*. *mansoni* diagnostics through the discovery of specific biomarkers or proteomic profiles associated with infection or disease stage. In addition to possible diagnostic advances, the application of MS techniques to serum samples from the well-described schistosome-infected mouse model also has the potential to provide novel insights into parasite biology. Although the choice of proteomic platform and the optimal timing of sampling were unknown at the launch of these studies, we hoped to identify candidate biomarkers at different time-points after infection, representing the different pathological stages of the disease: ie: EARLY prior to egg production (~3 weeks post-infection), ACUTE: ~6 weeks post-infection when eggs are being starting to be deposited in the liver, and CHRONIC: ~12 weeks post-infection when there is a well-defined granulomatous reaction in the liver.

Because several different MS platforms are available, each with its particular strengths and weaknesses, we opted to explore three complimentary approaches. We used high throughput surface-enhance, laser-desorption and ionization, time-of-flight mass spectrometry (SELDI-TOF MS) to compare uninfected and infected sera as a ‘proof-of-principal’ exercise. We subsequently used sample fractionation and differential gel electrophoresis prior to analysis on two more precise MS platforms; specifically matrix-assisted, laser-desorption and ionization (MALDI-TOF MS) and Velos Orbitrap MS. By using multiple proteomic platforms in parallel, we demonstrated that serum protein profiles differ extensively between infected and uninfected mice, offering a rich source of potential biomarkers.

## Materials and Methods

### Mouse infection and serum collection

Twenty-six female CD1, six-week old mice were purchased from The Charles River (St. Constant, Québec). All animal experiments were approved by the Facility Animal Care Committee of McGill University and followed the guidelines of the Canadian Council on Animal Care. Mice (5/group) were infected intraperitoneally (IP) with 50, 100, 150 or 200 *S*. *mansoni* cercariae (*S*. *mansoni*-infected *Biomphalaria glabrata* snails were obtained from the Biomedical Research Institute; Bethesda, MD). Control animals (n = 6) were IP injected with PBS. Mice were maintained in ventilated cages and monitored once per week. Blood samples were collected by saphenous bleeding before infection, at three weeks and six weeks post-infection. Mice were sacrificed by CO_2_ narcosis and blood was collected by direct cardiac puncture at 12 weeks post-infection. All sera were kept at −80°C until analyzed.

### Fractionation method

We used sample fractionation prior to SELDI and MALDI analysis. Serum samples at different time points were fractionated as previously described [10], [11]. Briefly, ProteinChip serum fractionation kit (Bio-Rad) was used to fractionate the samples into six pH fractions prior to mass spectrometric analysis. 96-well Q-Ceramic HyperD F resin filtration plates were used for the fractionation. To decrease the complexity, 20 μL of sample were first incubated with 30 μL of U9 buffer (9 M urea 2% CHAPS, and 50 mM Tris-HCl, pH 9) for 20 minutes. Then, samples were diluted with 50 μL of U1 buffer [1 M urea, 2% (wt/vol) 3-[(3-cholamidopropyl)-dimethylammonio]-1-propanesulfonate (CHAPS), 50 mM Tris-HCl, pH 9] and added to pre-equilibrated filtration plates incubated on a MicroMix machine (MicroMix 5; Diagnostic Products Company, Los Angeles, CA) for 30 minutes. Five fractions were collected by serial elution in buffers of decreasing pH (pH9, 7, 5, 4, 3). The buffers used are wash buffer 1 (50 mM Tris-HCl, 0.1% OGP, pH 9), wash buffer 2 (50 mM HEPES, 0.1% OGP, pH 7), wash buffer 3 (100 mM Na acetate, 0.1% OGP, pH 5), wash buffer 4 (100 mM Na acetate, 0.1% OGP, pH 4) and wash buffer 5 (50 mM Na citrate, 0.1% OGP, pH 3). After the collection of the first five fractions, a final elution by vacuum in an organic buffer was collected. The buffer used in the final collection was wash buffer 6 (33.3% isopropanol, 16.7% acetonitrile, 0.1% trifluoroacetic acid). All fractions were stored at -80°C until used.

### SELDI-TOF MS

SELDI-TOF MS was introduced by Hutchens T.W. and Yip T.T in 1993 [12] and has many potential advantages including high throughput, small volume requirement, a broad dynamic range and the capacity to analyze complex samples such as serum by pre-fractionation on chromatographically-distinct ‘chips’. The fractionated serum samples were bound to ProteinChip™ arrays (Bio-Rad) and analyzed essentially as previously described [10], [11]. Cation Exchange (CM10) and Immobilized Metal Affinity Capture (IMAC30) Protein-Chip™ arrays were used to profile the fractionated samples as previously described [11]. Each chip type was read under low and high laser intensity (2500 and 3750 nanojoules respectively). Biomarker Patterns Software™ algorithms (Bio-Rad) were then used to analyze the peak intensities and to generate classification ‘trees’ by CART analysis (Bio-Rad Laboratories) [13].

### ProteinChip Data Manager Software

ProteinChip Data Manager Software (version 3.5; Bio-Rad) was used first to determine protein clustering. The latter defines a group of peaks of similar mass that are treated as the same protein or peptide across multiple spectra. The peaks generated within an MS spectrum indicate signal intensity on the y-axis and mass-to-ion ratio (*m*/*z*) on the x-axis. The same peptide or protein peaks with different levels from each serum sample were collected and grouped into clusters. Two-step spectral analysis was performed. In the first pass analysis, automated detection identified peaks using a signal-to-noise ratio (S/N) of 5, and cluster peaks with a *p*-values ≤ 0.05 were inspected and relabeled manually. In the second pass analysis, peak detection was performed on the user-defined peaks only, and peaks with S/N ratio ≥ 2 were retained. The Biomarker Wizard application of the ProteinChip™ software was then used to compare the data between control and infected groups using the Mann–Whitney test. Potential biomarkers were defined as peaks with p-values of ≤ 0.05 and Receiver Operation Characteristic (ROC) values of < 0.3 and > 0.7.

### Classification and regression tree [CART] Analysis

Biomarker Pattern Software™ (BPS) (Bio-Rad Laboratories) was used to analyze the SELDI peak intensities and generate classification trees (i.e., potential diagnostic algorithms). Using multiple differentially-expressed protein peaks in the sera of infected and control groups, the BPS software creates a series of decision-points at cut-off intensities assigned to achieve the best separation. Sera with values <X go to the left node while those with values ≥X go to the right node. The software repeats this splitting process for each daughter node using other candidate biomarker peaks until terminal nodes are produced [13]. CART analysis of SELDI data has been used, among other applications, to identify candidate diagnostic biomarkers in Chagas disease [11], to differentiate hepatocellular carcinoma patients from cirrhotic subjects [14], and for diagnosis of lung adenocarcinoma [15].

### SDS-PAGE and in-gel digestion

The fractionated pooled serum samples (1 μL/lane) from control (12 week) as well as acutely (6 week) and chronically infected mice (12 week) were loaded onto 4–12% Bis-TrisNuPAGE gels (Invitrogen Life Technologies, Carlsbad, CA), and run for 45 min at 200 V. After which the gel was with silver stain (silver staining kit; Bio-Rad). Gel images were captured by scanning and then comparing the intensities of stained spots to compare the amount of specific proteins visually and certain spots were selected for analysis. Gel bands of differentially-expressed proteins (n = 45) were excised.

### Protein identification by mass spectrometry

Prior to MS analysis all the gel pieces were dried under the vacuum and then rehydrated in Trypsin Digest Solution (proteomics grade, Roche) at 37°C overnight. Peptides were eluted in a total volume of 5 μl of acetonitrile and 0.1% trifluoro acetic acid solution (50:50 v/v), after desalting with Millipore's C18 Zip Tips, the samples were resuspended in 10 μL 0.1% TFA and subjected to MALDI MS analysis.

### MALDI-TOF MS

MALDI-TOF MS is a powerful technique that has been used for protein identification from different biological samples [16]. The resulting digests were analyzed by 4800 Plus MALDI-TOF/TOF analyzer ABSciex (Applied Biosystems, Foster City, CA), as previously described [11]. Briefly, the samples were eluted in alpha-cyano-4-hydroxycinnamic acid matrix (Sigma-Aldrich, St Louis, MO). One μl aliquots was spotted directly onto a 384-well Opti-TOF MALDI stainless steel plates (AB Sciex, Framingham, MA) and allowed to air-dry at room temperature. Ions were generated by pulsing the mixture with a nitrogen laser (Laser energy at 4000 nanojoules). The collected spectra were analyzed using ProteinPilot™ software (Applied Biosystems) by searching the mouse and *S*. *mansoni* Swiss-Prot protein databases. The enzyme of cleavage was trypsin, fixed modification was carbamidomethyl, and variable modification was oxidation. The confidence threshold for protein identification was set to 95%.

### LTQ-Orbitrap Velos

LTQ-Orbitrap mass analyzers can analyze thousands of proteins with high resolution, and high mass accuracy. This approach is useful for identification of unknown proteins even at low concentrations in complex body fluids [17]. We used LTQ-Orbitrap Velos analysis primarily to pursue schistosomal antigens in the infected sera (Clinical Proteomics Platform, McGill University). One μL of pooled serum from control (12 weeks), acutely (6 weeks) and chronically infected mice (12 weeks) were loaded onto precast 10% mini-protean TGX precast gels (BioRad, Mississauga, ON) and run for 45 min at 200 V. The protein gel spots were visualized by staining with Coomassie blue gels (BioRad) for at least 1 h and destained with 10% acetic acid for 30 min. Twenty horizontal bands were excised from each lane, placed in 1.5 mL tubes and exposed to 40 μL of 100 mM NH_4_HCO_3_ for 5 minutes then centrifuged for 10 min at 14,000 × g, and 4°C. After aspiration of the liquid, the gel pieces were vortexed with 40 μL of 100 mM NH_4_HCO_3_/50% acetonitrile—for 10 minutes to shrink the gel. The gel pieces were dried in a microplate at 60°C in a vacuum concentrator for 30 minutes to remove excess acetonitrile and then placed in 40 uL of 10 mM dithiothreitol (DTT)/100 mM NH_4_HCO_3_ (Sigma-Aldrich, St Louis, MO) at 56°C in a closed water bath for 60 min. Gel pieces were then alkylated in 40 uL of 55 mM iodoacetamide + 100 mM NH_4_HCO_3_ for 45 min in 1.5 mL tubes in the dark at room temperature. After centrifugation at 14,000 x g for 15 min, the fluid was aspirated and gel pieces were washed in the same 1.5 mL tube in 100 uL of 100 mM NH_4_HCO_3_. Tubes were then centrifuged at 14,000 x g for 10 min, followed by removal of the fluid and washing of the gel in 40 uL of 100 mM NH_4_HCO_3_ for 5 min at room temperature after which 40 uL of acetonitrile (ACN) was added to make 1:1 solution and incubation was continued for 15 min at room temperature. This last wash-step with 100 uL of 25mM NH_4_HCO_3_ in 50% ACN was repeated one time then the gel pieces were dried (60°C as above) and digested with 10–20 uL (enough to cover pieces) of trypsin (Roche, Mannheim, Germany) overnight at 37°C (12.5 ng/μL). After digestion, peptides were extracted with 100 μL 1% formic acid and vortexing at 37°C for 15 min. Tubes were then centrifuged at 14,000 x g for 15 min, and the supernatants were transferred to new 1.5 mL tubes (first extraction). A second extraction was performed with 100 μL 5% formic acid/50% acetonitrile, (Sigma-Aldrich, St Louis, MO) and, after centrifugation (14,000 x g for 15 minutes), the two extraction supernatants were pooled. The pooled supernatants were then dried in a vacuum centrifuge (Hermle Labortechnik, Germany) for ~1–2 hours at 50°C. Dried samples were re-suspended in 40 uL 0.05% formic acid and stored at −20°C until used for MS analysis.

### Western Blots

Neat sera samples (1 μL/lane) from control (12 weeks) and acutely (6 weeks) or chronically infected mice (12 weeks) were loaded onto 4–12% Bis-TrisNuPAGE gels (Invitrogen Life Technologies, Carlsbad, CA), and run for 45 min at 200 V. Isolated proteins were transblotted onto nitrocellulose membrane (Invitrogen, KiryatShmona, Israel) at 100 V for seven min and then stained with Ponceau S (Sigma-Aldrich) to verify transfer and as an initial loading control [18], [19]. Membranes were blocked (5% skim milk in 0.05% Tween 20 in PBS: PBST) buffer for 1 hour at room temperature (RT), followed by three washes with PBST for 5 min each. Membranes were incubated overnight at 4 °C with the following antibodies: (1) mouse anti-α1-antitrypsin monoclonal antibody (1:500—Pierce Biotechnology, Rockford, IL) (2) rabbit anti-transferrin polyclonal antibody (1:500—Santa Cruz Biotechnology, Dallas, Texas) (3) rabbit polyclonal antibody against *S*. *japonicum* GST (1:500—US biological, Salem, MA) (4) mouse anti-actin monoclonal antibody (1:000—Santa Cruz Biotechnology). The membranes were washed three times with PBST for 5 min each and incubated with horseradish peroxidase-labeled respective anti-mouse, anti-rabbit, and anti-mouse secondary antibodies (Amersham Biosciences Co., Piscataway, NJ) diluted in 5% non-fat dry milk in PBST (1:10000) for 1 h at room temperature. The membranes were washed one time in PBST for 15 minutes followed by 2 washes for 5 minutes each then incubated in Super Signal West Pico detection solution (Pierce, Rockford, IL) and exposed to X-ray film. The ratio between the targeted proteins and the actin control bands were used to standardize across samples [20], [21]. Glyceraldehyde-3-phosphate-dehydrogenase (GAPDH) was used as an internal loading control. Membranes were stripped with 100 ml of stripping solution: 2% sodium dodecyl sulfate (SDS), 62.5 mM tris HCL pH 6.7, 100 mM mercaptoethanol for 30 minutes at 55°C followed by re-probing with a mouse anti-GAPDH monoclonal (1:5000: Abcam, Toronto, ON). Blots were incubated with HRP-conjugated anti-mouse IgG at 1:10,000 at RT for 1 h (Amersham Biosciences Co.) and exposed to X-ray film. Image J software (National Institutes of Health, Bethesda, MD) was used to analyze densities of selected bands.

## Results

### Screening of serum biomarkers in early, acute, and chronic stages of *S*. *mansoni* infection

In the samples analyzed by SELDI-TOF-MS, a total of 66 candidate biomarkers corresponding to peptides and proteins between 2.9 kDa and 82.3 kDa were identified at the three time points (versus control sera). This experiment suggested that the greatest number of potential biomarkers were present in fractions 1 and 6 (F1, F6).

**Fig 1:**
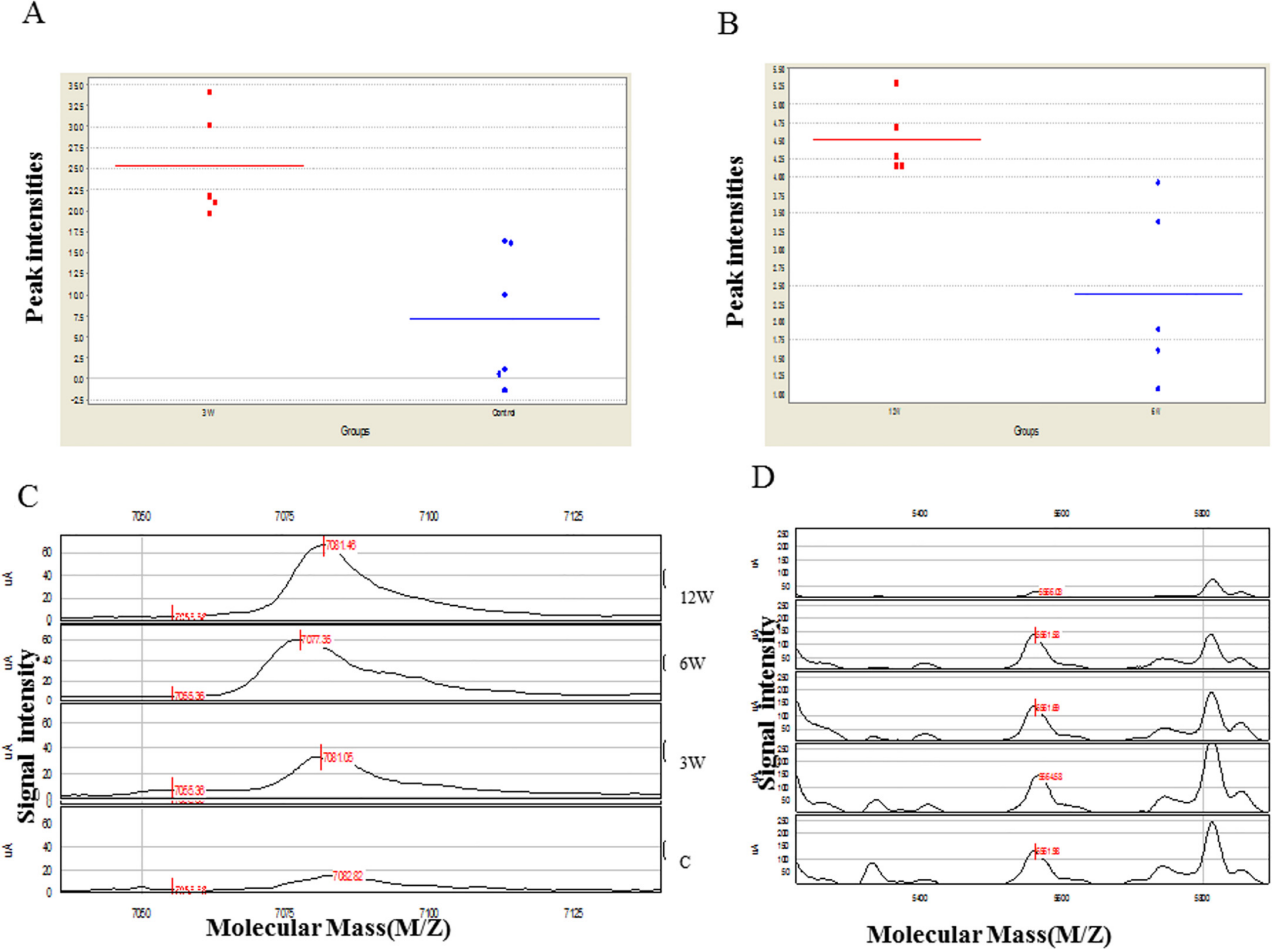
Examples of candidate biomarkers. (A) Scatterplot showing the discrimination of the early infection and control groups using m/z 3720 at F6ISL (**F**raction **6**, **IMAC** chip, **L**ow laser intensity). Blue dots: control. Red dotes: early stage of infection. (B) Scatterplot showing the discrimination of the acute and chronic groups using m/z 13407.2 at F6CSH (**F**raction **6**, **CM10** chip, **H**igh laser intensity). Blue dots: acute. Red dotes: chronic. (C) Example of a candidate biomarker increased in infected mice over time. Serum SELDI-TOF MS mass spectra obtained for F6ISL (**F**raction **6**, **IMAC** chip, **L**ow laser intensity) from infected mice (top 3 spectra) versus non-infected mice (bottom spectrum). A candidate biomarker as 7081 Da is gradually up-regulated from 3, to 6, to 12 weeks after infection (P value 0.006). (D) Example of a candidate biomarker increased in infected mice regardless the parasite burden. Serum SELDI-TOF MS mass spectra obtained for F6CSL (**F**raction **6**, **CM10** chip, **L**ow laser intensity) from infected mice (bottom 4 spectra) versus non-infected mice (top spectrum). A candidate biomarker as 5566.31 Da is up regulated in infected mice dependent on the infection rather than the dose of infected agents (P value 0.006). G1 infected with 200 cercariae, G2 infected with 150 cercariae, G3 infected with 100 cercariae, G5 infected with 50 cercariae.

All were statistically significant (p-value ≤ 0.05) with ROC (Receiver Operation Characteristic) curve values either 0.3< or >0.7. Among the 66 differentiating peaks, a 3720 Da protein (p< 0.006) was one of the most interesting and potentially useful. The SELDI data indicated that 3720 Da protein was highly over-expressed in early infection (versus control: Fig 1A) making it a potential candidate for identifying the early stage of schistosomiasis. A second biomarker at 13,407 Da (p< 0.01) reliably differentiated acute from chronic infection (6 versus 12 weeks: Fig 1B). A representative example of discriminatory peaks presented in Fig 1C proved that the signal intensity of the peak increased with the prolonged time of infection. Another representative example of discriminatory peaks presented in Fig 1D proved that the signal intensity of the peak increased in all different groups regardless of the parasite burden.

### Detection and validation of SELDI peaks

**Fig 2:**
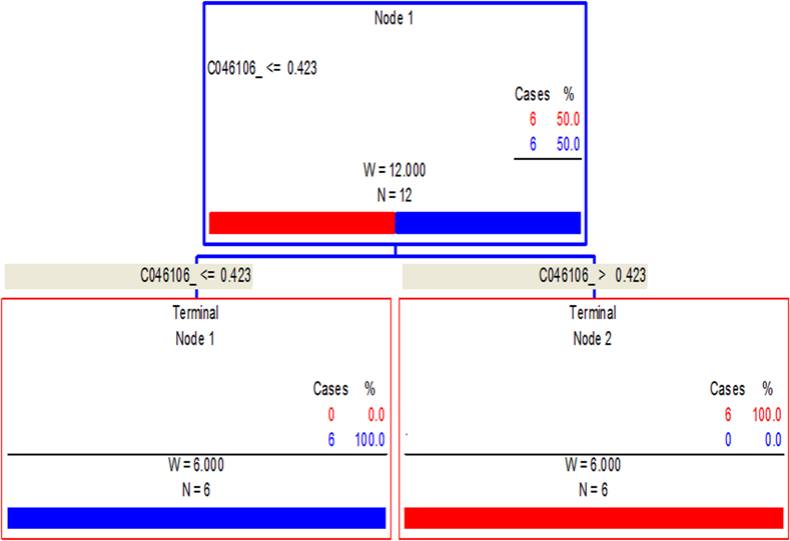
Biomarker pattern software based on CART analysis. A nonparametric procedure, was used to generate candidate diagnostic algorithms. The CART based on a series of binary decision trees that recursively partition a data set into blocks of predicted positive and negative samples. The CART procedure pursues to minimize a cost function that balances prediction errors in false-positive or false-negative results as well as the total number of biomarkers used. An example of decision tree classification using infected (G1) vs controls is shown. In this algorithm, the intensities of the 46106 Da biomarker establish the splitting rules. The samples have an intensity of ≤0.423 are placed in the left daughter node, and samples that have an intensity of ≥0.423 go to the right daughter node. Terminal red boxes = uninfected; blue boxes = acute infection.

BPS™ was then used to analyze the peak intensities by generating classification trees from all of the SELDI data. For example, Fig 2 shows a candidate diagnostic algorithm for acute infection based upon the IMAC array biomarker of 46 kDa. This single biomarker decision tree achieved 100% sensitivity and 100% specificity.

### Biomarker identification

The identification of candidate biomarkers is crucial to their translation into useful clinical and scientific ‘tools’. Unfortunately, the SELDI-TOF-MS platform does not permit peptide/protein identification, yielding only approximate molecular weight ‘peaks’. To identify these peaks we used two different approaches: peptide ‘fingerprinting’ with MALDI-TOF and peptide sequencing with Orbitrap.

### MALDI-TOF MS

We next used the MALDI-TOF for MS analysis of 1-dimensional SDS-PAGE gel slices of pooled sera from each group at different time points: 3, 6, and 12 weeks post infection. This approach generated eight potential biomarkers, all of host origin. Among these candidate biomarkers, serotransferrin and alpha 1-antitrypsin (AIAT) were the most convincing, as the former was identified with 12 peptides and the latter was identified with four peptides. These data suggest that these two proteins are present in infected mouse serum at relatively high abundance (Table 1).

**Table 1:** Eight proteins were increased in the sera of mice during both the acute and chronic stages of schistosomiasis compared to controls.

N	Unused	Total	%Cov	%Cov(50)	%Cov(95)	Accession	Name	Species	Peptides (95%)
1	3.36	3.36	28.16	28.16	28.16	tr|E9Q223|E9Q223_MOUSE	Hemoglobin subunit beta-1	MOUSE	4
3	2.01	2.01	9.091	4.924	4.924	tr|Q8BPD5|Q8BPD5_MOUSE	Apoa1 protein	MOUSE	2
1	20	20	12.58	12.58	12.58	tr|E9Q035|E9Q035_MOUSE	Serotransferrin	MOUSE	12
3	2	2	5.217	5.217	5.217	sp|Q91X72|HEMO_MOUSE	Hemopexin	MOUSE	2
2	0	2	2.138	2.138	2.138	tr|Q546G4|Q546G4_MOUSE	Albumin 1	MOUSE	1
3	2.56	2.56	7.089	7.089	4.557	tr|Q9DBN0|Q9DBN0_MOUSE	ApolipoproteinA-IV	MOUSE	1
3	2	2	5.327	5.327	5.327	tr|Q91X22|Q91X22_MOUSE	Alpha-1-antitrypsin	MOUSE	4
1	2	2	6.849	6.849	6.849	tr|Q9QUN8|Q9QUN8_MOUSE	Beta-2-globin	MOUSE	4

N: The rank of the specified protein relative to all other proteins in the list of detected proteins.

Unused (ProtScore): A measure of the protein confidence for a detected protein, calculated from the peptide confidence for peptides from spectra that are not already completely “used” by higher scoring winning proteins.

Total (ProtScore): A measure of the total amount of evidence for a detected protein. The Total ProtScore is calculated using all of the peptides detected for the protein.

% Cov (Coverage): The percentage of matching amino acids from identified peptides having confidence greater than 0 divided by the total number of amino acids in the sequence.

% Cov (50): The percentage of matching amino acids from identified peptides having confidence greater than or equal to 50% divided by the total number of amino acids in the sequence.

% Cov (95): The percentage of matching amino acids from identified peptides having confidence greater than or equal to 95% divided by the total number of amino acids in the sequence.

Accession #: The accession number for the protein.

Peptides (95%): The number of distinct peptides having at least 95% confidence.

### Velos Orbitrap

The Orbitrap analysis was performed with mouse sera from acute and chronic stages of infection to pursue parasite antigens as promising candidates. Here we provide, for the first time, the identity of the set of schistosomal proteins detected from mouse serum by Velos Orbitrap mass spectrometry that show great promise as potential biomarkers. Scaffold revealed 597 and 454 proteins from samples of the acute and chronic mice sera, respectively. This number was reduced to 200 and 105 proteins by requiring 95% probability and the presence of at least one identified peptide (Tables A and B in S1 File). Scaffold also revealed 654 and 774 host proteins from the acute and chronic mice sera, respectively. As previously outlined, this number was also reduced significantly to 296 and 220 proteins by imposing the same conditions for acceptance (Tables C and D in S1 File).

These parasite-origin proteins may reflect proteins released by damage to the tegument, or secretory/excretory products released by worms into mouse serum. It has been suggested that confidence in protein identification is increased by replication and independent identification of the same proteins [22], [23]. In order to increase confidence in sensitive Orbitrap data, the Orbitrap data sets have been recorded in and compared (in replicated experiments). Confidence also increases with the number of peptides identified from each protein [22], [23]. Therefore, we filtered the results by Validation Category of Spectrum Mill Software using number of identified spectra, distinct peptide numbers, % amino acid (%AA) coverage, and total protein spectral intensity. Using Spectrum Mill Software, we brought the number of parasite-origin protein down to 28 proteins in both acute and chronic stages (Table 2). Among these 28 candidate biomarkers, actin was the most frequently-identified protein. Ryanodine receptor-related protein was identified with a distinct summed MS/MS search score of 15.36, %AA coverage of 0.1, and total protein spectral intensity of 1.97E+09. Protocatechuate dioxygenase was also identified with a distinct summed MS/MS search score of 12.95, %AA coverage of 3.3, and total protein spectral intensity of 4.74E+06. Most proteins were identified with only one peptide however, which may be explained by the low concentrations of the parasite antigens compared to the host antigens in the serum of infected animals. Most of the protein identifications were based on single peptides putting their identification in doubt without additional confirmatory studies. When available, the classical approach for confirmation is the running of a Western blot. We performed Western blotting for GST (one of the proteins identified with only one peptide) and confirmed the MS identification.

**Table 2:** Shows these proteins ordered by Spectrum Mill; generally the higher abundant identified proteins.

Spectra	Distinct peptide	Distinct Summed MS/MS Search score	% AA Coverage	Total Protein Spectral intensity	Database Accession number	Protein Name
223	10	157.6	14.1	6.00E+11	15808978	albumin precursor
11	7	108.9	23.1	9.59E+08	2.56E+08	Actin
13	1	15.36	0.1	1.97E+09	2.56E+08	ryanodine receptor related
1	1	13.17	5.9	6.85E+06	2.56E+08	rap1 and
1	1	12.95	3.3	4.74E+06	2.56E+08	protocatechuate dioxygenase
1	1	12.67	10.1	1.88E+06	2.83E+08	MHC class I antigen
1	1	12.57	24.4	1.37E+09	2.56E+08	hypothetical protein
1	1	12.41	2.1	2.07E+08	2.56E+08	hypothetical protein
1	1	11.74	4.4	1.32E+08	76152891	SJCHGC08921 protein
3	1	11.71	2	6.13E+08	2.56E+08	calcium binding protein-related
1	1	11.59	5	7.07E+06	2.56E+08	elongation factor 1-alpha (ef-1-alpha)
1	1	11.25	0.8	7.41E+06	2.56E+08	hypothetical protein
1	1	11.05	6.3	3.67E+07	2.56E+08	mitochondrial phosphate carrier protein
1	1	10.7	0.7	8.17E+06	2.56E+08	lyst-interacting protein
1	1	10.66	3.7	3.52E+08	76155947	SJCHGC04200 protein
1	1	10.53	2.3	9.41E+06	2.56E+08	hypothetical protein
1	1	10.51	3.7	5.85E+06	2.56E+08	glycerol-3-phosphate dehydrogenase
1	1	10.49	5.6	2.84E+09	76152647	SJCHGC05123 protein
1	1	10.36	7.9	3.56E+07	76162212	SJCHGC01996 protein
1	1	10.35	3.1	1.36E+07	2.56E+08	hypothetical protein
1	1	10.29	6.8	6.26E+07	2.57E+08	Tropomyosin-2
1	1	10.28	0.4	8.04E+07	2.56E+08	hypothetical protein
1	1	10.26	3.4	3.38E+06	2.56E+08	heat shock protein
1	1	10.24	0.7	2.82E+07	2.56E+08	protein kinase
1	1	10.19	10.7	1.46E+08	76152933	SJCHGC03943 protein
1	1	10.11	1.9	9.85E+06	2.56E+08	myotubularin-related protein
1	1	10.02	0.2	5.19E+08	2.56E+08	hypothetical protein
1	1	10.01	3.2	2.72E+06	2.56E+08	tegumental protein

### Overlap between the three mass spectrometry platforms

Although SELDI-TOF provided proof-of-principle that serum from mice with acute and chronic infection had distinct protein patterns (from each other and from control animals), this approach could not identify the differentially expressed proteins. To overcome this limitation, we turned to the MALDI-TOF and Orbitrap platforms to identify the differentially expressed proteins. In our hands, MALDI-TOF had relatively limited sensitivity, identifing only eight host proteins over-expressed in the infected mice (haemoglobin beta, apolipoprotein A-I, serotransferrin precursor, hemopexin, serum albumin precursor, apolipoprotein A-IV precursor, alpha-1-antitrypsin, and beta-globin). All eight were also identified as up-regulated in infection by Orbitrap (100% probability) and all but hemopexin were reflected in corresponding SELDI peaks (±5% mass range) when the SELDI database was queried. Of the 66 candidate biomarker peaks identified by SELDI as differentially-expressed (either up- or down-regulated), all but 20 (30.3%) were tentatively identified in the Orbitrap database (±5% mass range) (data not shown). The consistency with which these candidate biomarkers were identified across the three different platforms strongly supports the validity of these observations. Overlap between the proteomic biomarkers on the three MS platforms are recorded in Table 3.

**Table 3:** Overlap between the three methods. This table shows the number of peaks detected in serum by MALDI and the overlap between them and the similar peaks detected by the other two methods Orbitrap and SELDI.

MALDI	Orbitrap		Orbitrap (acute stage)		Orbitrap (chronic stage)		SELDI	
Name	Accession N.	molecular weight	Peptides (95%)	AA coverage	Peptides (95%)	AA coverage	molecular weight	M/Z Average
Hemoglobin subunit beta-1	1183933	16 kDa	6	89%	1	96%	16297.84	F6ISL
Apoa1 protein	231557	31 kDa	27	77%	23	71%	34996.4	F6ISH
Serotransferrin	20330802	77 kDa	65	73%	52	83%	82364	F6CSH
Hemopexin	15030012	51 kDa	28	64%	23	61%		
Albumin 1	163310765	69 kDa	65	88%	58	85%	64508.29	F1ISH
Apolipoprotein A-IV	110347473	45 kDa	26	74%	20	63%	46278.31	F6ISH
Alpha-1-antitrypsin	6678085	46 kDa	4	47%	21	60%	46573.27	F1ISH
Beta-2-globin	156257619	16 kDa	3	93%	2	86%	16297.84	F6ISL

### Western Blot

**Fig 3:**
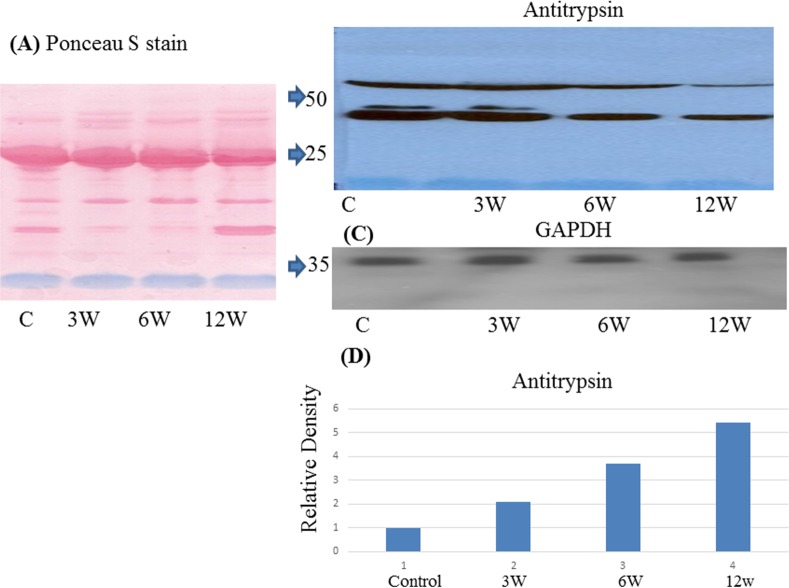
Immunologic validation of A1AT as a candidate biomarker. (A) Representative Western blot of A1AT in pooled sera from *S*. *mansoni*-infected mice (6 and 12 weeks post-infection) and controls. (B) Ponceau stained gel and (C) Western blot of GAPDH served as a loading controls. (D) Relative density of A1AT proteins levels normalized to GAPDH.

**Fig 4:**
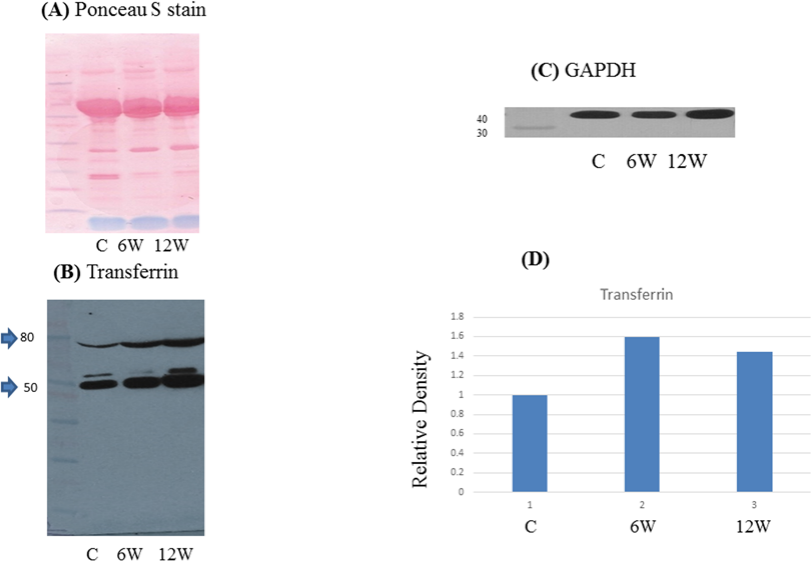
Immunologic validation of transferrin as a candidate biomarker. (A) Representative Western blot of transferrin in pooled sera from *S*. *mansoni*-infected mice (6 and 12 weeks post-infection) and controls. (B) Ponceau stained gel and (C) Western blot of GAPDH served as a loading controls. (D) Relative density of transferrin proteins levels normalized to GAPDH.

To confirm the accuracy of the MALDI and Orbitrap analyses, we performed Western blots for some of the identified proteins. Both A1AT (alpha 1-antitrypsin) and serotransferrin were differentially expressed between infected and control serum samples (Figs 3 and 4). For AIAT, the whole protein (~50 kDa) was present but the antibody recognized two bands; the upper band corresponding to full-length A1AT while the lower band (~25 kDa) is likely an A1AT fragment. Western blotting also confirmed that transferrin is highly expressed in the sera of infected mice, the whole protein (~79 kDa) was detected but the antibody recognized two bands; the upper band at 79 kDa while the lower band at 50 kDa is likely a transferrin fragment.

**Fig 5:**
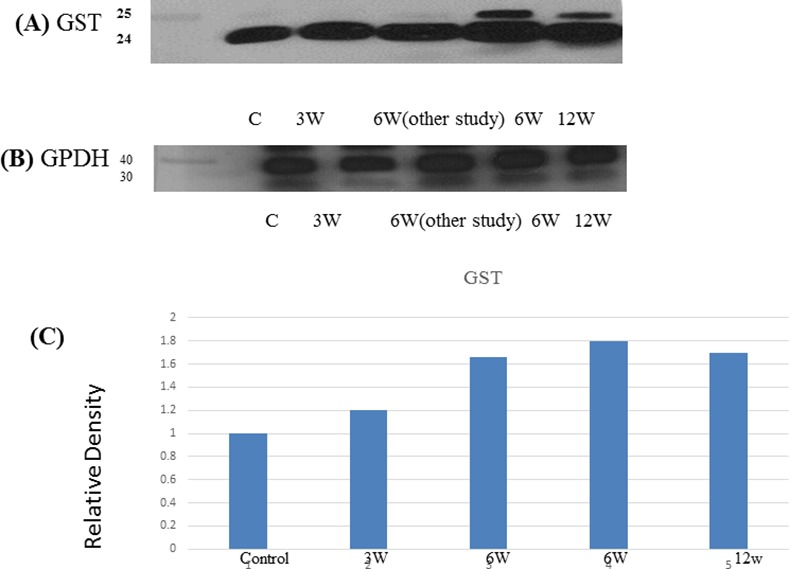
Immunologic validation of GST as a candidate biomarker. (A) Representative Western blot of transferrin in pooled sera from *S*. *mansoni*-infected mice (3, 6 and 12 weeks post-infection as well as sample from 6 weeks post infection from different study) and controls. (B) Western blot of GAPDH served as a loading controls. (C) Relative density of GST proteins levels normalized to GAPDH.

**Fig 6:**
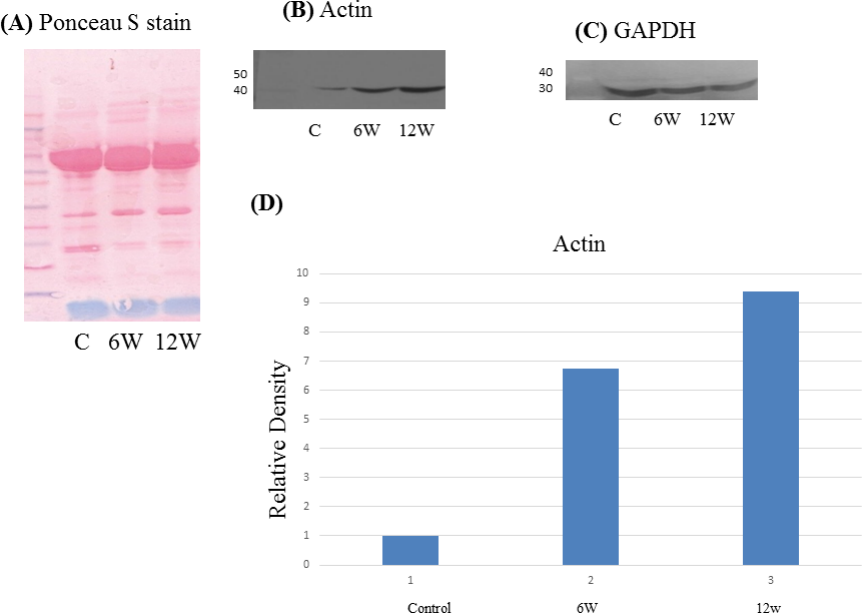
Immunologic validation of actin as a candidate biomarker. (A) Representative Western blot of actin in pooled sera from *S*. *mansoni*-infected mice (6 and 12 weeks post-infection) and controls. (B) Ponceau stained gel and (C) Western blot of GAPDH served as a loading controls. (D) Relative density of actin proteins levels normalized to GAPDH.

Orbitrap data suggested the presence of both mouse (~24 kDa) and schistosome (~25 kDa) GST, which was confirmed by Western blot. The intact whole protein (molecular weight ~24 kDa) was present in control sera while, the antibody recognized two bands in acutely and chronically-infected animals; the upper band (molecular weight ~25 kDa) corresponds to the schistosome GST while the lower band (~24 kDa) likely corresponds to the mouse GST (Fig 5). Orbitrap data also suggested the presence of schistosome actin (42 kDa), which was confirmed by Western Blot. The antibody recognized one band (42 kDa) in the sera of control and infected mice, both in acute and chronic stages (Fig 6).

## Discussion

In 2003, a large transcriptome database that contained resources for peptide searches for *S*. *mansoni* was published [24]. The publication of the *S*. *mansoni* genome provided approximately 17,250 full-length predicted genes [25]. Protasio and colleagues [26] had reduced the number of predicated genes to 10,852, using Sanger capillary and deep-coverage Illumina sequencing from clonal *S*. *mansoni* worms. A recent version of the *S*. *mansoni* genome was published, using the next-generation sequencing (NGS) technology [27]. These genomic tools provide invaluable resources for protein identification.

There is an urgent need to provide early detection techniques of schistosomiasis so that the infection can be eliminated before egg deposition and complications occur. Given that SELDI-TOF MS is relatively high-throughput, requires minute sample for analysis, we chose this technique as our ‘first-line’ approach for biomarker discovery. Using SELDI, we were able to identify several candidate biomarkers with promise as markers of early-stage infection. While the SELDI platform provided ‘proof-of-concept’, these data were restricted to biomarker ‘peaks’ rather than identified proteins.

In order to identify some of the SELDI biomarker peaks, we exploited the MALDI-TOF and Velos Orbitrap platforms. Serum transferrin and glycoprotein A1AT were identified by MALDI and confirmed by Western blot. Transferrin levels were increased in both acute and chronic stages of disease. Transferrin is a ‘negative’ acute phase reactant that is synthesized in the liver as a glycoprotein and is involved in iron transport [28]. Serum levels are increased in alcoholic fatty liver disease [29] but decreased in hepatic diseases that suppress synthetic capacity [30]. In human and murine schistosomiasis, serum transferrin levels have varied substantially between studies. For example, our finding are consistent with those of Salawu and Arinola (2004) [31] who reported increased serum levels in urinary schistosomiasis. Harvie et al. (2007) [32] also reported increased liver transferrin levels in C57BL/6 mice after 8 weeks of *S*. *mansoni* infection. These same authors found elevated serum transferrin in chronic hepatosplenic schistosomiasis in CBA/J mice and suggested that this protein might be potential biomarker for hepatosplenic disease [33]. In contrast, Saif et al. [34], Arinola [35] and Arinola & Salimonu [36] have reported decreased serum transferrin levels in hepatosplenic and urinary schistosomiasis in a sample of patients in Egypt and Nigeria, respectively. Plasma transferrin levels tend to increase in patients suffering from iron deficiency anemia [37] and Mansour & Farid [38] have shown an association between *S*. *mansoni* infection and anemia. Given these previous findings from different studies, we postulate that increased levels of transferrin could be a result of gastrointestinal blood loss and iron deficiency anemia that is associated with schistosomiasis.

Four A1AT peptides were identified using the MALDI platform, A1AT is a member of the serpin family (serine protease inhibitors) and acts as a trypsin inhibitor. It is synthesized as a single polypeptide chain of about 51 kDa mainly in the liver but also in macrophages and epithelial cells [39]. Deficiency of this protein is associated with many liver diseases such as neonatal hepatitis [40], cirrhosis and hepatoma [41]. Finally, this protein is considered a potential biomarker for hepatitis B virus infection [42]. The serpin family has been reported to increase in inflammation [43]. In our murine schistosomiasis model, A1AT was increased in both the acute and chronic stages of the infection, both of which are associated with significant inflammatory responses.

Surprisingly, the Velos Orbitrap identified a large number of putative parasite-origin proteins in serum of infected mice. The large number of schistosome proteins in the serum from the *S*. *mansoni* infected mice may be attributable, in part, to our use of the serum from the later time-points (6 and 12 weeks post-infection) in the Orbitrap analysis. This strategy may have allowed schistosome proteins to accumulate in the blood after being secreted, shed from live parasites, or released upon parasite destruction mediated by the host immune system [44], [45], [46]. Many of the schistosome proteins that we found in low concentrations in the serum of the infected mice are potentially interesting in understanding the biology of schistosomiasis. However, the more abundant proteins are likely to be the most useful for clinical applications. We therefore sought to prioritize the more abundant proteins for further validation using Validation Category of Spectrum Mill Software. Some of these proteins were known to be present in the parasite tegument including (a) actin, which was the most abundantly identified protein with 7 identified peptides and 23.1% amino acid coverage; (b) tegumental protein, with 1 identified peptides and 3.2% amino acid coverage and (c) a 14 KDa calcium-binding protein of unknown function. The tegument of *S*. *mansoni* is limited by two lipid membrane bilayers that contain several spines [47], [48]. These spines are composed mainly of actin bundles [49]. Using fluorescence microscopy, actin has been present on the surface of schistosomula [50]. In addition, actin proved to be present in the muscle, tegumental tubercles and spines of male and female adult parasites using immunofluorescence [51]. Actin has been implicated in the maintenance of the integrity of the tegument of the adult worm, either associated with the spines, or free in the cytoplasm [52]. Our finding of actin in the serum of the infected mice might be the result of shedding from the schistosomula released from dying worms or damaged to the tegumental cell layer.

We also identified released enzymes such as glutathione S-transferase (GST) that play a crucial role in defence against oxidative stress [53]. Heat shock protein (HSP60) was also identified as an abundant protein in this proteomic analysis. By looking for commercial antibodies for one of these potential biomarkers we found one against *S*. *japonicum* GST. GST enzyme family uses glutathione in reactions contributing to the detoxification of endogenous and exogenous toxins [54]. Increased levels of host GST have been reported in hepatocellular carcinoma [55] and schistosomiasis [35]. However, Manivannan, et al. reported decreased levels of GST in *S*. *mansoni* infected mice [33]. In our study, we found overexpression of GST in acute and chronic stages of *S*. *mansoni* infected mice. The overexpressed GST reacted with anti-GST antibody of *S*. *japonicum*. This suggests that the GST originated from the parasite as identified by Velos Orbitrap. Therefore, GST can be considered as an infection-associated antigen and its overexpression may be considered as a potential serum biomarker for the early diagnosis of *S*. *mansoni*.

The identified biomarkers cannot be applied as a routine diagnostic test in their current format, and require experts to translate the potency of these biomarkers into a rapid, accurate, easy to use and cheap (i.e. <$2) field test. While this study assessed candidates in a small and well-defined mice study, future studies with patient populations will help to validate the usefulness of these candidates as biomarkers of schistosomiasis. It is not easy to regularly monitor serum proteomic changes during the course of infection in human. Identification of these proteins in human could be more difficult because of the low concentration of the parasite antigens compared to the host antigens in the serum of infected patients.

## Summary and Conclusion

Large numbers of candidate host- and parasite-origin biomarkers for acute and chronic schistosomiasis in mice were identified using a combination of SELDI, MALDI and Orbitrap technologies. We found little overlap in the biomarkers identified (based on m/z); most appeared to be unique. These three platforms, therefore, performed in a complementary fashion in our biomarker discovery program. Our program revealed that serum protein profiles differ extensively between infected and uninfected mice and between the early, acute and chronic stages of infection in mice, offering a rich source of candidate biomarkers. If similar differences are identified in human disease, this approach may not only yield new diagnostic strategies but may also give insights into parasite biology and point to novel targets for treatment or prevention.

## Supporting Information

S1 File List of candidate protein biomarkers whose were found in the mice sera. List of candidate schistosomal protein biomarkers whose were found in the acute mice sera (Table A). List of candidate schistosomal protein biomarkers whose were found in the chronic mice sera (Table B). List of candidate host protein biomarkers whose were found in the acute mice sera (Table C). List of candidate host protein biomarkers whose were found in the chronic mice sera (Table D).
(XLSX)
